# Geometry-aware view reconstruction network for light field image compression

**DOI:** 10.1038/s41598-022-26887-4

**Published:** 2022-12-23

**Authors:** Youzhi Zhang, Lifei Wan, Yifan Mao, Xinpeng Huang, Deyang Liu

**Affiliations:** 1 School of Computer and Information, Anqing Normal University, Anqing, 246000 China; 2 University Key Laboratory of Intelligent Perception and Computing, Anqing Normal University, Anqing, 246003 China; 3 School of Communication and Information Engineering, Shanghai University, Shanghai, 200444 China; 4 Key Laboratory of Specialty Fiber Optics and Optical Access Networks, Shanghai University, Shanghai, 200444 China

**Keywords:** Computer science, Information technology

## Abstract

Light Field (LF) imaging empowers many attractive applications by simultaneously recording spatial and angular information of light rays. In order to meet the challenges of LF storage and transmission, many view reconstruction-based LF compression methods are put forward. However, occlusion issue and under-exploitation of LF rich structure information limit the view reconstruction qualities, which further influence LF compression efficiency. In order to alleviate these problems, in this paper, we propose a geometry-aware view reconstruction network for LF compression. In our method, only sparsely-sampled LF views are encoded, which are further used as priors to reconstruct the un-sampled LF views at the decoder side. The proposed reconstruction process contains two stages including geometry-aware reconstruction and texture refinement. The geometry-aware reconstruction stage utilizes a multi-stream framework, which can fully explore LF spatial-angular, location and geometry information. The texture refinement stage can adequately fuse such rich LF information to further improve LF reconstruction quality. Comprehensive experimental results validate the superiority of the proposed method. The rate-distortion performance and the perceptual quality of reconstructed views further demonstrate that the proposed method can save more bitrate while increasing LF reconstruction quality.

## Introduction

Light field imaging can encode 3D scene information into 4D LF images by simultaneously recording spatial and angular information of light rays^1^. The additional angular information enables many attractive applications, such as depth estimation^2,3^, 3D reconstruction^4^, post-capture refocusing^5^, and virtual/augmented reality^6,7^, et al. Especially, with the development of handheld plenoptic camera (i.e., Lytro^8^ and RayTrix^9^), LF imaging technique has drawn a wide research interest.

**Figure 1:**
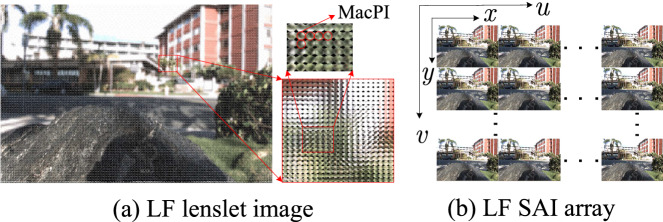
LF lenslet image and SAI array representations. The two LF representations can transform each other, and can be regarded as an equivalent representation.

The 4D LF can be model by a two-plane parameterization model, which can be described as $L=L(u,v,x,y)$, where (*u*, *v*) denotes the two angular dimensions and (*x*, *y*) represents the two spatial dimensions. Based on the 4D representation, LF has two main visualization forms, i.e., LF lenslet image and Sub-Aperture Image (SAI) array, as shown in Fig. 1. The LF lenslet image can be considered as a 2D collection of Macro-Pixel Images (MacPIs). By extracting pixels at the same spatial coordinates of each MacPI and organizing them together, we can obtain a SAI (also referred to as view). Therefore, the LF lenslet image and SAI array can be regarded as an equivalent representation.

The high-dimensional LF structure produces a large volume of data, which brings great challenges for LF storage and transmission. For instance, one decoded LF image captured by handheld plenoptic camera Lytro Illum needs around 50 MB storage space. The bulky data becomes the main bottleneck for LF imaging development. Therefore, developing high-efficiency LF image compression methods is of great importance for LF applications.

Many researchers have focused on LF image compression^10,11^. For example, in Feb. 2015, JPEG committee launched a project (referred to as JPEG Pleno^12^) aiming at the standardization of LF image compression. Based on two LF representations, the LF compression methods can be divided into two categories: lenslet image based and SAI array based. The lenslet image based LF compression methods^13–17^ try to improve LF compression efficiency by exploring the correlations between the neighbouring MacPIs under the existing image/video coding standards. While the SAI array based methods^18–24^ intent to enhance the compression performance by eliminating redundancies of adjacent SAIs. Wherein, based on the wide applications of Convolutional Neural Network (CNN) in LF image processing, learning based LF view reconstruction methods^25–31^ are introduced into the LF compression. The main idea of learning based LF compression method is to encode sparsely-sampled LF SAIs at the encoder side and synthesize the rest of SAIs with learning based view reconstruction at the decoder side. Since the learning based LF compression method can remove more LF spatial and angular redundancies, it becomes the mainstream technology for LF image compression. However, two limitations still exist for learning based LF compression. Firstly, since occlusion breaks the photo consistency assumption^32^, artifacts are inevitably introduced around the occlusion regions during view reconstruction, which reduces the qualities of reconstructed views. Even though many LF view reconstruction solutions^33–37^ are put forward to suppress occlusions, abundant LF spatial, angular and geometry characteristics are still not fully considered. As a result, the reconstruction performance is not encouraging. Therefore, mitigating occlusion problem is still the key issue to further enhance the compression performance. Secondly, exploiting LF spatial, angular and geometry information benefits in recover more texture details of reconstructed views. However, most existing methods overlook adequate fusion of such information, which limits the compression performance.

In order to alleviate these problems, in this paper, we propose a geometry-aware view reconstruction network for LF compression. Different from some existing LF view reconstruction methods, the proposed method entirely explores LF spatial and angular correlations and fully fuse LF spatial-angular, location and geometry information for high-quality view reconstruction. Moreover, in order to suppress occlusion issues, we explicitly estimate the disparity map of 4D LF from decoded sparse SAIs to model scene geometry. The proposed framework contains two stages including geometry-aware reconstruction and texture refinement. Specifically, the geometry-aware reconstruction stage adopts a multi-stream framework including three modules, i.e., disparity estimation module, view synthesis module, and 3D Deconv module. The disparity estimation module utilizes the disparity-based warping paradigm, where a 4D disparity map is estimated firstly, and then a dense LF stack is synthesized by warping the input SAIs based on the estimated 4D disparity map. The view synthesis module can reconstruct a dense LF by exploring the LF spatial and angular information with a proposed view synthesis network. 3D convolution operations are applied in the proposed view synthesis network to allow an information propagation between LF spatial and angular dimensions. 3D Deconv module considers the dense LF reconstruction by utilizing a decovolution layer, where the SAI location information is explored to keep the consistency of reconstructed SAIs. The texture refinement stage introduces a view refinement network, which takes full use of 3D convolution operations to adequately fuse the three output dense LFs from geometry-aware reconstruction stage to restore more texture details. The main contributions of this paper are as follows:

- This paper proposes to suppress occlusion issues occurred during LF reconstruction process by explicitly model scene geometry. Moreover, we propose to fully fuse LF spatial-angular, location and geometry information for high-efficiency LF compression.
- We construct a geometry-aware view reconstruction network for LF image compression. The proposed reconstruction process contains two stages including geometry-aware reconstruction and texture refinement. Our network allows an adequate interaction of LF spatial-angular, location and geometry information, which benefits in recovering more texture details during LF reconstruction.
- Comprehensive experimental results demonstrate the superiority of the proposed method in improving LF compression efficiency as compared to some state-of-the-art methods.

The rest of this paper is organized as follows. In “Related work” section, the related works of LF compression are reviewed. In “Proposed method” section, we introduce the proposed LF compression network. The simulation results and analyses are given in “Experimental results” section. We conclude the proposed method in “Conclusion” section.

## Related work

The bulky data severely influences the applications of LF imaging. Therefore, many researchers have devoted to developing high-efficiency LF compression methods. This section will briefly review the existing LF compression solutions including lenslet image based methods and SAI array based methods.

### Lenslet image based methods

Since the lenslet image can be seen as a collection of 2D MacPIs which correlate strongly, the lenslet image based compression methods explore to improve LF compression performance by reducing the correlations within and across MacPIs. For example, Monteiro et al.^13^ utilized the spatial redundancy among neighbouring MacPIs, and proposed a two stage intrablock prediction method under High Efficiency Video Coding (HEVC) standard^38^ to predict each image block. Conti et al.^14^ proposed a scalable LF coding solution, where a self-similarity (SS) prediction mode was introduced to HEVC standard to improve the compression performance. Jin et al.^15^ put forward a macropixel-based intra prediction method for LF compression, in which three modes including multi-block weighted prediction mode, co-located single-block prediction mode, and boundary matching-based prediction mode were adopted to fully explore spatial correlations of LF lenslet image. Subsequently, for plenoptic 2.0 videos, Jin et al.^39^ proposed a novel compression method to further improve intra prediction performance by integrating two new intra prediction modes including imaging-principle-guided static prediction and imaging-principle-guided zoomed prediction. Considering the strong correlations of lenslet image, Liu et al.^16^ introduced a Gaussian process regression (GPR) based prediction mode to HEVC to improve intra prediction accuracy. However, the GPR based prediction mode was sensitive to the correlations among the adjacent coding blocks, and the computation complexity was high both at the encoder and decoder sides. To alleviate these issues, Liu et al.^17^ further put forward a content-based LF compression method, where the prediction units were divided into three categories including non-homogenous texture units, homogenous texture units, and visually flat units. Three different prediction methods were respectively utilized for different categories, which acquired a high prediction accuracy. In order to remove redundancies of lenslet image, Liu et al.^40^ introduced the 4D Epanechnikov mixture regression into LF compression, where a 4D Epanechnikov mixture regression and a linear function-based reconstruction were adopted for high-efficiency LF compression.

The lenslet image based LF compression methods exploit the strong correlations existed among neighbouring MacPIs to enhance compression performance. However, the prediction accuracy becomes the main bottleneck for further LF compression performance improvement. Moreover, the lenslet image based methods overlook the rich angular information of LF image, which also influences the compression efficiency. Therefore, many efforts are devoted to SAI array based LF compression, which will be revisited in the next subsection.

### SAI array based methods

LF SAI array can be extracted from LF lenslet image, and can reflect more LF spatial and angular correlations. Many researchers have focused on LF compression based on LF SAI array representation. The SAI array based methods can be further divided into three categories: pseudo sequence based method, prediction based method, and learning based LF reconstruction method.

#### Pseudo sequence based methods

The pseudo sequence based methods propose to arrange the SAIs into a pseudo-video sequence, and apply intra- and inter prediction modes of existing image/video coding standards to improve compression efficiency. For instance, Liu et al.^18^ proposed a pseudo sequence based LF compression method. By investigating the coding order of SAIs, prediction structure, and rate allocation, the compression efficiency was dramatically improved. Dai et al.^19^ firstly arranged the LF SAIs into a pseudo sequence with Line Scan Mapping (LSM), and then encoded the arranged pseudo sequence with the HEVC standard. Mehajabin et al.^41^ put forward an efficient pseudo-sequence-based LF video coding method, in which two prediction structures and coding orders were utilized. In order to further remove redundancies, disparity-based warping paradigm is introduced in pseudo sequence based method. The sparse SAIs and the disparity maps corresponding to the unsampled SAIs are encoded at the encoder side. Disparity-based warping is utilized to synthesize the unsampled SAIs at the decoder side. For example, Jiang et al.^20^ put forward a LF compression scheme based on depth-based view synthesis technique, in which a subset of SAIs were compressed at encoder side and then were used to reconstruct the entire LF with depth-based warping at the decoder side. Huang et al.^21^ focused on LF structural consistency, and proposed a low bitrate LF compression method. In their method, a color-guided refinement algorithm and a content-similarity-based arrangement algorithm were proposed to keep the geometry and content consistencies. Even though the disparity-based LF compression can remove more redundancies, the qualities of synthesized SAIs are susceptible to the quality of estimated disparities.

#### Prediction based methods

By exploiting the 4D structure of LF, prediction based methods are utilized for high-efficiency LF compression. For example, Alves et al.^12^ reported the design and integration of the first LF codec (i.e., MuLE) based on 4D-DCT, in which two new coding modes were adopted including 4D-Transform mode and 4D-Prediction mode. By using a binary-tree-oriented bit-plane clustering and a 4D-native hexadeca-tree-oriented bit-plane clustering, the compression performance was improved. Astola et al.^22^ put forward a LF coding method by using disparity-based sparse prediction. In their method, a new inter-view residual coding method and a region-based sparse prediction method were adopted to further increase the LF coding efficiency. Miandji et al.^23^ put forward a dictionary learning based LF compression method, in which a multidimensional dictionary ensemble (MDE) was trained to sparsely represent LF data. Moreover, they also introduced a nonlocal pre-clustering approach to construct an aggregate MDE to reduce the training time. Santos et al.^42^ studied three 4D prediction and partition modes for LF lossless encoding including an hexadeca tree, a 4D partition using a quadtree, and a conventional 2D partition with multiple references. Chao et al.^43^ described a LF coding method based on graph based lifting transform, where redundancies and distortions were suppressed during raw data compression process. Ahmad et al.^24^ divided the SAIs into two classes including key views and decimated views. The key views were compressed using MV-HEVC method, while the decimated views were synthesized by utilizing a shearlet-transform-based prediction scheme at the decoder side. By sending the residual information of synthesized SAIs to the decoder side, their method achieved a better compression efficiency under low bitrates.

#### Learning based LF reconstruction method

The learning based LF reconstruction method aims to learn a mapping from decoded sparse SAIs to dense LF at the decoder side to improve the LF compression performance. For example, Chen et al.^25^ proposed to firstly learn a disparity-based global representation, and was then used to predict the decimated SAIs. Jia et al.^26^ presented a LF compression framework by using a Generative Adversarial Network (GAN)-based SAI synthesis technique, where a multi-branch fusion network, a refinement generative network and a discriminative network were integrated to enhance the qualities of reconstructed SAIs. Hou et al.^27^ taken full use of inter- and intra-view correlations of LFIs, and proposed a LF compression method with bi-level view compensation. In their method, the learning based SAI synthesis was adopted to compensate the reconstructed SAIs, while a block-wise motion compensation was utilized to ensure the coding efficiency. Wang et al.^28^ put forward a LF compression scheme with multi-branch spatial transformer networks based view synthesis. In order to generate high-quality target unselected SAIs, a multi-branch spatial transformer network was designed to learn the affine transformations of neighboring SAIs. Bakir et al.^29^ designed a dual discriminator GAN to synthesize the dropped SAIs at the decoder side. Subsequently, this work was improved in^30^ by adding a multi-view quality enhancement network to ensure the reconstruction qualities of synthesized SAIs. Liu et al.^31^ constructed a multi-disparity geometry structure of sparse SAIs at the decoder side and then put forward a multi-stream view reconstruction network to reconstruct the entire LF. By exploring the abundant LF geometric structure information, this method achieved a high compression performance. Zhang et al.^44^ presented a LF compression method by leveraging graph learning and dictionary learning. In their method, the learned graph adjacency matrix was used to synthesize the dropped SAIs, and then the dictionary-guided sparse coding was developed to encode such graph adjacency matrices. Tong et al.^45^ proposed to decouple the spatial and angular information using dilation convolution and stride convolution and jointly compress the decoupled spatial and angular information with a feature fusion network. For dynamic LF video compression, Hu et al.^46^ firstly down-sampled the LF video with a multi description separation method, and then used a GNN-based LF compression method to decode and recover all the descriptions.

The learning based LF reconstruction method only needs to encode sparse SAIs at the encoder side and synthesizes the entire SAIs with learning based view reconstruction at the decoder side. With more and more delicate networks are designed, the LF compression performance has been continuously improved. However, the occlusion problem and the under-exploitation of LF spatial, angular and geometry information still limit the reconstruction qualities of synthesized SAIs, which further influences the LF compression performance. In this paper, we construct a geometry-aware view reconstruction network for LF image compression, in which the disparity map of 4D LF is explicitly estimated to suppress occlusion issue and LF spatial, angular and geometry information are fully fused for high-quality reconstruction.

**Figure 2:**
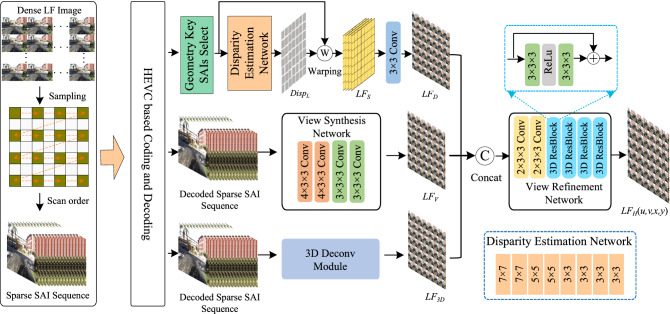
The flowchart of the proposed LF compression method. The dense LF SAIs are firstly sparsely-sampled and compressed at the encoder side. The un-sampled SAIs are synthesized at the decoder side with the proposed geometry-aware view reconstruction network, which contains geometry-aware reconstruction and texture refinement stages.

## Proposed method

The flowchart of the proposed LF image compression method is described in Fig. 2. At the encoder side, the dense LF SAIs are firstly sparsely-sampled. The sample process is illustrated in Fig. 2. For a $7\times 7$ dense LF, the proposed method only needs to compress $4\times 4$ sampled SAIs at the encoder side, and the other un-sampled SAIs are synthesized at the decoder side. Similar to the most existing learning based LF reconstruction method, the sparsely-sampled SAIs are arranged into a pseudo-sequence according to a specific scan order which is shown in Fig. 2. HEVC standard is utilized to encode the obtained pseudo-sequence. At the decoder side, the decoded sparse SAIs are fed into the proposed geometry-aware view reconstruction network to synthesize a dense LF image.

The entire view reconstruction process contains two stages including geometry-aware reconstruction and texture refinement. The geometry-aware reconstruction aims to mine LF spatial-angular, location and geometry information to suppress occlusion issues, while the texture refinement tires to restore more texture details by fully fuse such LF spatial-angular, location and geometry information. Note that, in our method, the proposed view reconstruction network is only applied on luma component (Y). The bilinear interpolation method is adopted for reconstructions of the chrominance components (Cb and Cr).

### Geometry-aware reconstruction

The geometry-aware reconstruction stage adopts a multi-stream framework, which intents to fully exploit LF spatial-angular, location and geometry information to achieve a high-quality LF reconstruction. The geometry-aware reconstruction stage contains three modules including disparity estimation module, view synthesis module, and 3D Deconv module. The detail network frameworks are illustrated in the next sub-sections.

**Figure 3:**
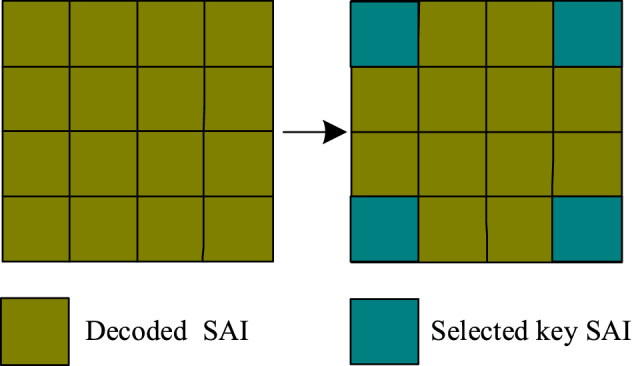
Geometry key SAIs selection. Only four corner SAIs in the decoded sparse SAI sequence are selected as the geometry key SAIs.

#### Disparity estimation module

The disparity estimation module explores to apply the disparity-based warping paradigm to mine LF geometry structure information for dense LF reconstruction. For a $7\times 7$ dense LF, there are $4\times 4$ SAIs in the decoded sparse SAI sequence. In order to reduce computational burden, a geometry key SAIs selection procedure is firstly adopted before disparity estimation. In this procedure, we only select the four corner SAIs in the decoded sparse SAI sequence as the geometry key SAIs to estimate the 4D disparity map, which is shown in Fig. 3. The selected geometry key SAIs are then fed into the disparity estimation network to obtain the 4D disparity map $Disp_{L}$. The disparity estimation network is consisted of two convolutional layers with kernel size $7\times 7$, two convolutional layers with kernel size $5\times 5$, and four convolutional layers with kernel size $3\times 3$. Every convolution layer is followed by a ReLU layer. After deriving the 4D disparity map, a disparity-based warping operation is conducted. For our case, four dense LFs can be obtained by warping four selected geometry key SAIs based on the estimated disparity map. Let $LF_{s} \in \mathbb {R}^{U\times V \times X\times Y \times C}$ denotes the derived dense LF stack, where (*U*, *V*) denotes the two high angular dimensions, (*X*, *Y*) represents the two spatial dimensions, and *C* refers to as the LF image channel. The acquired $LF_{s}$ is subsequently fed into a 2D convolutional layer with kernel size $3\times 3$ to generate one dense LF $LF_{D}$ by reducing the LF image channel from four to one. The disparity estimation based LF reconstruction process can be described as:

1 $$\begin{aligned} LF_{D}=f_{R}(\textbf{Warp}(SAI_{GK},Disp_{L})), \end{aligned}$$

where $f_{R}(\cdot )$ denotes the 2D convolutional operation to decrease the dimensions of LF image channel, $\textbf{Warp}(\cdot )$ is the backward warping operation, and $SAI_{GK}$ represents the four selected geometry key SAIs.

#### View synthesis module

The view synthesis module intents to synthesize a dense LF by fully exploring LF spatial and angular information. Since the neighbouring SAIs correlate strongly, it is advantage to reduce artifacts by adequately exploiting the correlations among the neighbouring SAIs. Therefore, in this paper, we design a view synthesis module to mine such correlations. In the proposed view synthesis module, the decoded sparse SAI sequence is directly fed into a view synthesis network to reconstruct dense LF. In order to explore the correlations among the neighbouring SAIs, 3D convolutional operations are utilized in the proposed view synthesis network, which enable information propagation between two spatial dimensions and one directional dimension of the decoded sparse SAI sequence. The proposed view synthesis network contains four 3D convolutional layers, in which the kernel sizes are $4\times 3 \times 3$, $4\times 3 \times 3$, $3\times 3 \times 3$, and $3\times 3 \times 3$, respectively. Note that, before being fed into the 3D convolutional layers, we reshape the tensor dimensions of decoded sparse SAI sequence from $({U\times V \times X\times Y \times C})$ to $({C\times UV \times X\times Y})$ to fit the 3D convolution operation. Moreover, for 3D convolution operation, the first dimension of 3D convolution kernel is for angular dimension, and the other two dimensions are for spatial dimensions. For example, for 3D convolution kernel $4\times 3 \times 3$, “$4$” is for angular dimension and “$3\times 3$” is for spatial dimensions. With the view synthesis module, we can derive a dense LF $LF_{V}$ which contains abundant LF spatial and angular structure information. The view synthesis process can be represented as:

2 $$\begin{aligned} LF_{V}=f_{V}(SAI_{DS}), \end{aligned}$$

where $f_{V}(\cdot )$ denotes the view synthesis network, and $SAI_{DS}$ is the decoded sparse SAI sequence.

#### 3D Deconv module

In LF reconstruction process, the location information of decoded sparse SAIs benefits in keep the consistency of reconstructed dense LF^47^. Therefore, we propose a 3D Deconv module to exploit the SAI position information as a supplement for dense LF reconstruction. The 3D Deconv module is formed by one 3D deconvolution layer with kernel size $7\times 1\times 1$, where “$7$” is for angular dimension and “$1\times 1$” is for spatial dimensions. With the 3D deconvolution layer, a dense LF $LF_{3D}$ can be obtained by upsamping the decoded sparse SAIs in angular domain. The dense LF $LF_{3D}$ can provide rich location information of decoded sparse SAIs for reconstruction. The 3D Deconv process can be expresses as:

3 $$\begin{aligned} LF_{3D}=f_{De}(SAI_{DS}), \end{aligned}$$

where $f_{De}(\cdot )$ denotes the 3D deconvolution operation, and $SAI_{DS}$ is the decoded sparse SAI sequence.

### Texture refinement

We obtain three dense LF $LF_{D}$, $LF_{V}$, and $LF_{3D}$ after the geometry-aware reconstruction stage. The $LF_{D}$ contains rich LF geometry information, $LF_{V}$ comprises abundant LF spatial-angular information, and $LF_{3D}$ provides the location information of decoded sparse SAIs. In order to fully exploiting these information to recover more texture details, we further propose a texture refinement stage. This stage enables a sufficient fusion of such information to synthesize a high-quality dense LF.

In this stage, the acquired three dense LFs are firstly concatenated together to form a dense LF stack $LF_{DS}\in \mathbb {R}^{U\times V \times X\times Y \times C_{S}}$, where $C_{S}$ denotes the LF image channel equaling to 3 in this paper. Subsequently, we further propose a view refinement network to fully fuse the rich LF spatial-angular, geometry, and location information to recover more texture details. The proposed view refinement network is consisted of two 3D convolutional layers with kernel size $2\times 3\times 3$, and four 3D ResBlock convolutional layers. Note that, the 3D ResBlock convolutional layer is consisted of two 3D convolutional layers with kernel size $3\times 3\times 3$, and one ReLu layer, which is shown in Fig. 2. Here, the first dimension of 3D convolution kernel is also for angular dimension, and the other two dimensions are for spatial dimensions. The texture refinement process can be expressed as:

4 $$\begin{aligned} LF_{H}(u,v,x,y)=f_{R}(\textbf{Concat}(LF_{D}, LF_{V}, LF_{3D})), \end{aligned}$$

where $LF_{H}(u,v,x,y)$ is the final reconstructed dense LF, $f_{R}(\cdot )$ denotes the view refinement network, $\textbf{Concat}(\cdot )$ represents the concatenation operation.

### Training details

In this paper, $\mathscr {L}_{1}$ loss is adopted to supervise our network by measuring distance between the synthesized dense LF $LF_{H}(u,v,x,y)$ and the ground-truth LF $LF_{GT}(u,v,x,y)$, which is represented as:

5 $$\begin{aligned} \mathscr {L}_{1}=\sum _{u,v,x,y}(|L_{GT}(u,v,x,y)-L_{HR}(u,v,x,y)|). \end{aligned}$$

We choose 83 LF images from the EPFL dataset^48^ to train our network. Moreover, 10 LF images are selected from the EPFL dataset according to the ICME Grand Challenge^49^ for testing. During training, every LF image is cropped into patches of size $64\times 64$ randomly in the spatial domain to construct the training dataset. The Adam optimizer^50^ is utilized to train our network. The initial learning rate is set to $1e^{-4}$, and the batch size is set to 1. The learning rate is decreased by a factor of 0.5 for every $1e^{3}$ epochs.

## Experimental results

In order to validate the efficiency of the proposed method, the proposed geometry-aware view reconstruction network is integrated into HEVC standard. At the encoder side, the sparse SAI sequence is firstly converted into YUV 420 format before compressed with HEVC. In our method, HEVC Test Model (HM) reference software version 14 with Low Delay P (LDP) coding configuration is utilized to encode the sparse SAI sequence. The proposed method is compared with four state-of-the-art LF compression methods. The abbreviations are listed as follows:

1. LSM^19^: The LF SAIs are arranged into a pseudo sequence with Line Scan Mapping (LSM), which is then encoded with HEVC standard.
2. PSC^18^: LF lenslet image is decomposed into multiple views which are then arranged into a pseudo sequence. The obtained pseudo sequence is then encoded with HEVC standard by considering the coding order, prediction structure, and rate allocation.
3. MuLE^12^: This method is the standard LF coding method which is provided by JPEG-Pleno. 4D-Transform mode is adopted in MuLE.
4. GCCM^21^: The sampled sparse SAIs and the corresponding disparities are encoded at the encoder side, and the un-sampled SAIs are synthesized at the decoder side with disparity based warping method.

The Bjontegaard metrics^51^ including BD-PSNR and BD-Rate are utilized to evaluate the performance of the proposed method.

**Figure 4:**
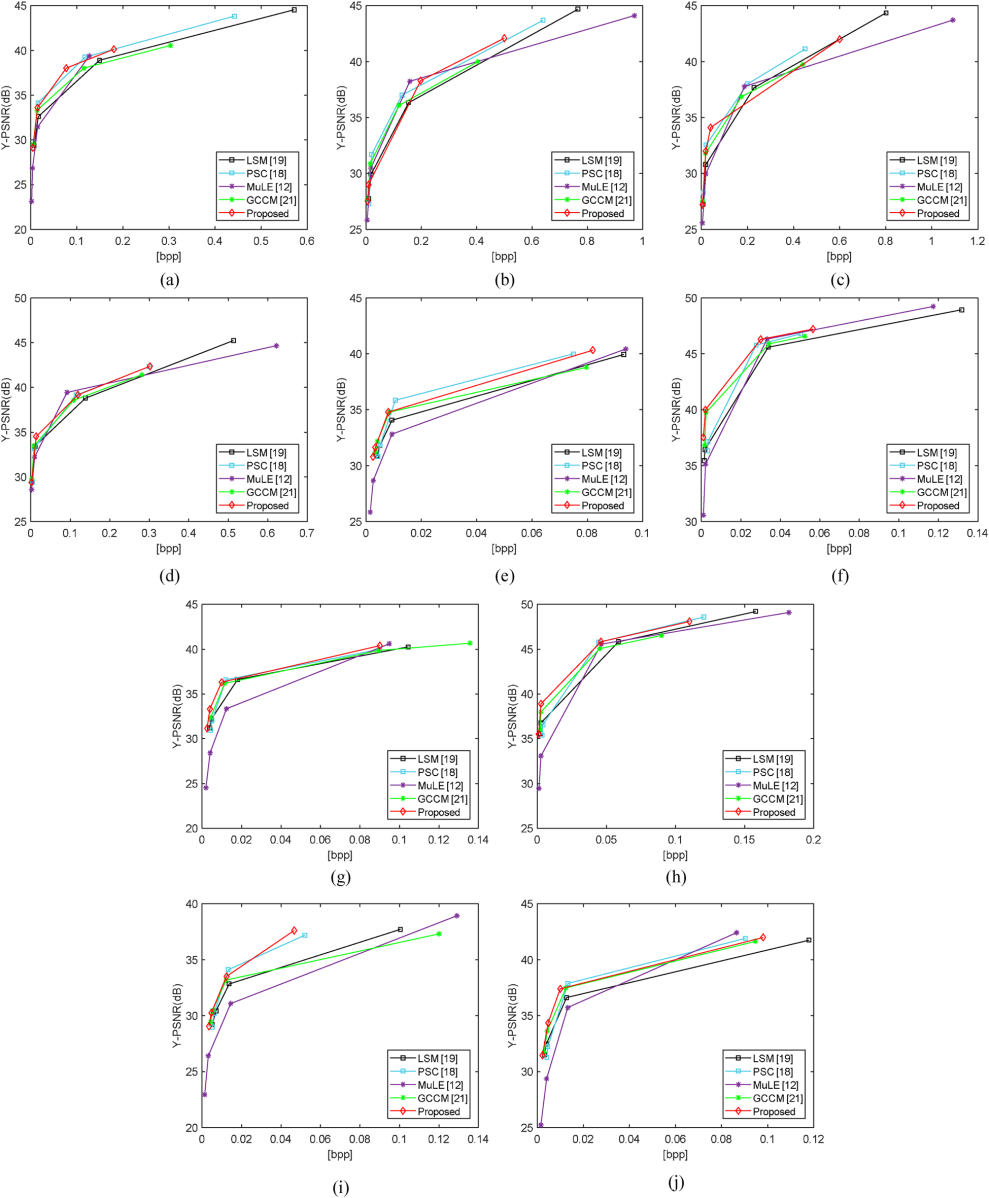
The RD curves for test LF scenes by using the LSM^19^, PSC^18^, MuLE^12^, GCCM^21^ and the proposed method. Average Y-PSNR and bits per pixel are adopted as the objective quality metrics: (**a**) I 01, (**b**) I 02, (**c**) I 03, (**d**) I 04, (**e**) I 05, (**f**) I 06, (**g**) I 07, (**h**) I 08, (**i**) I 09, (**j**) I 10.

**Figure 5:**
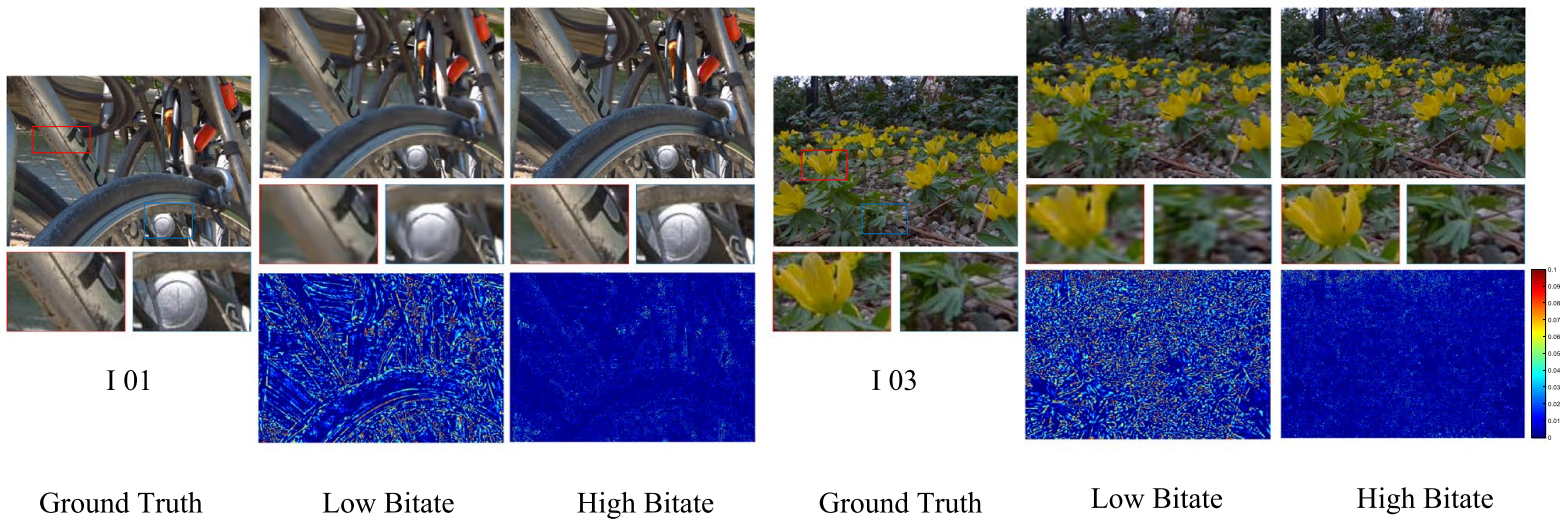
Perceptual quality comparisons of reconstructed SAIs for high and low bitrates by using the proposed method.

### Compression performance and analyses

The RD performance comparison of our method versus four state-of-the-art methods on Y channel are shown in Table 1. From Table 1, we find that the proposed method achieves the best average RD performance. Compared with LSM^19^, an average 0.93 dB BD-PSNR gain is derived by the proposed method. When compare to PSC^18^, the average gain of BD-PSNR is also over 0.25 dB. The main reason is that LSM and PSC only consider to construct a pseudo-video sequence by arranging the LF SAIs, and use intra- and inter prediction modes of HEVC to compress the LF data. However, a high degree of LF redundancies still exist during prediction process, which reduces the compression efficiency. The proposed method can reduce more LF redundancies and reconstruct high-quality LF views. As a result, the proposed method can obtain a higher compression efficiency. When compared with MuLE^12^, we can find from Table 1 that the average BD-PSNR gain is 1.78 dB. Especially for some LF image with complex texture, i.e., I 09, the BD-PSNR gain is 2.77 dB. It is because that the MuLE utilized 4D DCT to compress LF image, in which four separable 1D DCT are used on two spatial and two angular dimensions. However, for complex texture areas, it is hard to achieve an accurate prediction, which influences the compression performance. GCCM method proposes to compress sparse LF SAIs and the corresponding disparity maps to reduce LF redundancies. However, this method is sensitive to the quality of estimated disparity maps. Hence, an average of 0.35 dB BD-PSNR gain and up to 0.76 dB BD-PSNR gain for LF image I 05 is achieved by our method compared with GCCM.

**Table 1:** Y-rate-distortion performance of the proposed method compared with four state-of-the-art methods.

LF images	Proposed vs. LSM^19^		Proposed vs. PSC^18^		Proposed vs. MuLE^12^		Proposed vs. GCCM^21^	
	BD-PSNR (dB)	BD-rate (%)	BD-PSNR (dB)	BD-rate (%)	BD-PSNR (dB)	BD-rate (%)	BD-PSNR (dB)	BD-rate (%)
I 01	0.96	− 27.22	0.49	− 10.30	1.63	− 39.75	0.50	− 14.53
I 02	1.43	− 34.66	0.03	− 6.31	0.46	− 10.11	0.24	− 5.99
I 03	0.73	− 21.24	0.02	− 2.20	0.84	− 22.99	0.17	− 4.95
I 04	0.78	− 27.18	0.04	− 10.93	0.39	− 14.53	0.27	− 10.92
I 05	1.02	− 29.81	0.12	− 5.46	2.43	− 49.90	0.76	− 17.77
I 06	1.08	− 36.36	0.36	− 28.55	1.81	− 34.31	0.25	− 26.91
I 07	0.87	− 28.96	0.74	− 8.96	2.75	− 54.28	0.46	− 15.06
I 08	0.66	− 31.48	0.29	− 33.69	3.00	− 47.90	0.11	− 22.16
I 09	1.05	− 31.59	0.11	− 5.32	2.77	− 58.42	0.74	− 23.00
I 10	0.74	− 24.09	0.25	− 5.32	1.75	− 35.50	0.04	− 1.50
Average	0.93	− 29.26	0.25	− 11.70	1.78	− 36.77	0.35	− 14.28

Figure 4 gives the rate-distortion curves of the ten test LF images. The results from Fig. 4 are consistent with Table 1. From Fig. 4, we can also find that our method surpasses the other methods at low bitrates. However, for some LF scene, i.e., I 05 and I 10, the superiority of our method is limited at high bitrates. The main reason lies in two aspects. One is that the high-frequency information of decoded SAIs is lost for low bit rate coding. Compared with the other four methods, the proposed method can restore more texture details by fully exploring LF spatial-angular, location and geometry information. The other is that high bit rate coding can reserve more details in decoded LF SAIs. Even though our method can recover more texture details in reconstructed SAIs, the superiority is limited.

Figure 5 gives the perceptual quality comparisons of reconstructed SAIs at low bitrate and high bitrate by using our proposed method. We can observe from Fig. 5 that more texture details can be recovered at high bitrate. This is because that the perceptual quality of decoded sparse SAI sequence is higher than that of low bit rate coding. And, with the increase of bitrate, the qualities of reconstructed SAIs improves. By using the high-quality decoded sparse SAI sequence as the prior, the proposed reconstruction network can synthesize a high-quality dense LF, since more LF spatial-angular and geometry information can be exploited. However, the quality improvement slows down with the increases of bitrate, which is consistent with Fig. 4. It is because that the reconstruction process also introduces artifacts, even though the quality of decoded sparse SAI sequence is high.

**Figure 6:**
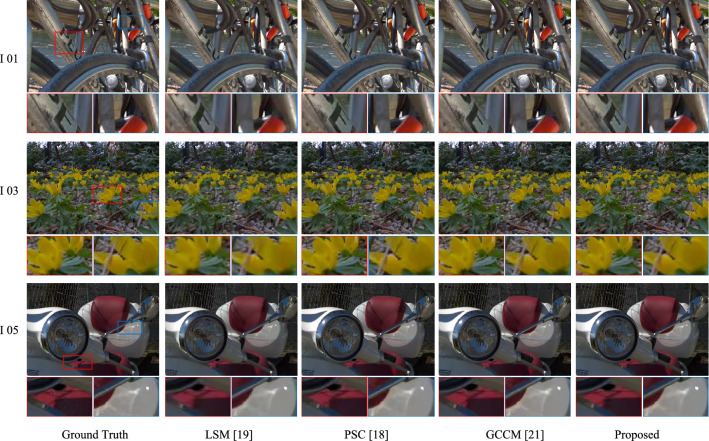
Perceptual quality comparisons of reconstructed SAIs at the decoder side at angular coordinate (4,4) at a similar bpp for three LF images, (i.e., 0.019 bpp for I 01, 0.03 bpp for I 03 and 0.017 bpp for I 05). The results show the ground truth SAIs at angular coordinate (4,4), the reconstructed SAIs by utilizing LSM, PSC, GCCM, and the proposed method, and the close-up versions of the image portions in blue and red boxes.

In order to further demonstrate the effectiveness of the proposed method, we compare our method with three other LF view reconstruction methods, i.e. Yang et al.^33^, Jin et al.^34^, and Yeung et al.^35^. We retrain the networks of the compared three methods to reconstruct $7\times 7$ dense LF images with $2\times 2$ sparse SAIs (four corner SAIs) as inputs. For fair comparison, we adjust our network, and also use four corner decoded SAIs as inputs to reconstruct $7\times 7$ dense LF images at the decoder side. 83 LF images from the EPFL dataset^48^ are used as the training data. The sparse views are encoded and transmitted to the decoder side, which are further utilized as the inputs to reconstruct dense LF image at the decoder side. The BD-Rate savings by our method versus the compared three methods is shown in Table 2.

**Table 2:** Y-rate-distortion performance of the proposed method compared with three LF reconstruction methods.

LF images	Proposed vs. Yang et al.^33^		Proposed vs. Jin et al.^34^		Proposed vs. Yeung et al.^35^	
	BD-PSNR (dB)	BD-rate (%)	BD-PSNR (dB)	BD-rate (%)	BD-PSNR (dB)	BD-rate (%)
I 01	− 0.11	8.83	0.19	− 14.02	0.11	− 8.60
I 02	0.10	− 5.20	0.39	− 24.70	0.27	− 17.14
I 03	0.30	− 20.58	0.53	− 32.54	0.38	− 25.48
I 04	0.17	− 11.44	0.67	− 37.44	0.38	− 23.31
I 05	0.22	− 6.14	0.83	− 37.64	0.58	− 28.75
I 06	− 0.25	18.36	0.34	− 22.90	0.09	− 4.81
I 07	0.01	− 0.95	0.15	− 14.58	0.09	− 14.11
I 08	− 0.04	4.20	0.56	− 48.44	0.07	− 5.98
I 09	0.04	− 2.97	0.18	− 15.38	0.11	− 12.16
I 10	− 0.15	16.52	0.30	− 26.91	0.20	− 18.51
Average	0.03	0.06	0.41	− 27.46	0.23	− 15.89

From Table 2, we find that the proposed method only achieves slight advantages when compared with the other three LF view reconstruction methods. The reasons lie in two aspects. One is that the proposed method can achieve a high view reconstruction quality by fully explore LF spatial-angular, location and geometry information. The other is that we adopt to explicitly exploit LF geometric disparity information to suppress occlusion issues.

### Perceptual quality of reconstructed SAIs

In order to further demonstrate that the proposed method can derive a high-quality reconstructed LF, we compare the perceptual quality of reconstructed SAI with angular coordinate (4,4) by using the proposed method with the decoded SAIs obtained by using LSM, PSC, and GCCM at a similar bpp, which is shown in Fig. 6. From Fig. 6, one can see that the proposed method achieves the highest perceptual quality than the other methods. For example, the results of LSM, PSC, and GCCM show more artifacts around the bicycle frame, flower petals, and motorcycle lamp than the proposed method. It is mainly because that the proposed method can remove more LF redundancies at the encoder side, which can save more bitrate than the other three methods. Moreover, the proposed method can adequately explore LF spatial-angular, location and geometry information, and reconstruct a high-quality dense LF by fully fusing such information.

### Ablation investigation

In order to investigate the effectiveness of the three modules of the proposed geometry-aware reconstruction stage, we conduct ablation experiments by respectively remove the disparity estimation module, view synthesis module, the 3D Deconv module, and the texture refinement module which are denoted as “w/o DE module”, “w/o VS module”, “w/o 3D Deconv module”, and “w/o TR module”. Table 3 gives the RD performance comparisons of our method versus the four variants on Y channel. From Table 3, we can find that the proposed method is obviously superior to the other three variants. Around average 0.14 dB, 0.13 dB, 0.81 dB, and 0.07 dB BD-PSNR gains are achieved by the proposed method versus the four variants. The main reasons lie in four aspects. Firstly, the disparity estimation module helps to mine LF geometry structure information for dense LF reconstruction. By explicitly modeling scene geometry with the disparity estimation module, the qualities of reconstructed SAIs are improved, especially for some occlusion regions. As a result, the compression performance is increased. Secondly, the view synthesis module fully explores LF spatial and angular information by using 3D convolutional operations. This benefits in restoring more texture details. By adding the view synthesis module, the reconstruction performance is enhanced, which further reinforces the LF compression efficiency. Thirdly, the 3D Deconv module can provide the location information of decoded sparse SAIs during reconstruction process. This helps to keep the consistency of reconstructed dense LF. Therefore, the compression performance improves by adding the 3D Deconv module. Fourth, the texture refinement module can fully fuse rich LF geometry information, LF spatial-angular information, and the location information of decoded sparse SAIs to recover more texture details. Therefore, by removing the texture refinement module, the reconstruction quality reduces, which further decreases the LF compression performance.

**Table 3:** Y-rate-distortion performance of the proposed method compared to four variants of our proposed method.

LF images	w/o DE module		w/o VS module		w/o 3D Deconv module		w/o TR module	
	BD-PSNR (dB)	BD-rate (%)	BD-PSNR (dB)	BD-rate (%)	BD-PSNR (dB)	BD-rate (%)	BD-PSNR (dB)	BD-rate (%)
I 01	0.19	− 5.13	0.21	− 5.81	0.90	− 21.86	0.11	− 3.27
I 02	0.07	− 1.83	0.08	− 2.24	0.92	− 21.73	0.10	− 2.69
I 03	0.07	− 2.10	0.08	− 2.38	0.64	− 16.65	0.06	− 1.81
I 04	0.04	− 1.57	0.03	− 1.21	0.62	− 18.75	0.04	− 1.43
I 05	0.21	− 6.57	0.22	− 6.86	0.66	− 18.71	0.12	− 3.99
I 06	0.15	− 4.96	0.09	− 3.00	0.98	− 25.60	0.03	− 1.13
I 07	0.32	− 11.64	0.28	− 10.35	0.69	− 22.86	0.11	− 4.14
I 08	0.16	− 5.17	0.09	− 2.98	0.99	− 26.37	0.04	− 1.31
I 09	0.06	− 2.10	0.08	− 2.60	0.77	− 22.39	0.04	− 1.15
I 10	0.13	− 4.24	0.11	− 3.38	0.93	− 25.05	0.04	− 1.39
Average	0.14	− 4.53	0.13	− 4.08	0.81	− 22.00	0.07	− 2.23

### Application on depth estimation

**Figure 7:**
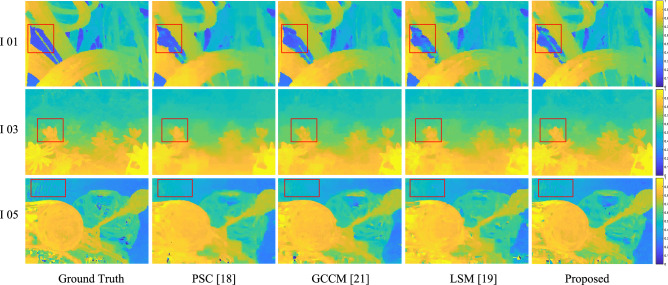
Quality comparisons of depth maps estimated from deocded LF image by using different methods at decoder side. The robust occlusion-aware depth estimation method^53^ is adopted to estimate the depth maps from decoded dense LF with angular resolution $7\times 7$.

High-quality dense LF can enhance the performance of scene depth estimation^52^. In order to further verify that the proposed method can reconstruct a high-quality dense LF with sparsely-sampled decoded SAIs as priors, we compare the qualities of estimated depth maps form decoded dense LF by using different methods, which is shown in Fig. 7. The robust occlusion-aware depth estimation method proposed in^53^ is adopted to estimate the depth map. From Fig. 7, we observe that a higher depth quality is obtained by using the proposed method. For example, the patches bounded by red boxes shows some estimated errors by using LSM, PSC, and GCCM. In contrast, our method can recover more depth details, and reduces estimated errors. This is mainly because the proposed method can recover more texture details during reconstruction process, which benefits in generating more depth details. However, compared with the ground truth depth map, one can further observe that some depth details are still lost in the estimated depth map by using the proposed method. This also demonstrates that the reconstruction procedure introduces distortions, especially for occlusion regions. Improving reconstruction quality is the key issue for LF compression and other LF applications.

## Conclusion

In this paper, a geometry-aware view reconstruction network is proposed for LF image compression. The dense LF is firstly sampled at the encoder side, and only sparsely-sampled SAIs are transmitted to the decoder side, which are further utilized as priors to reconstruct the rest SAIs. The reconstruction process contains two stages including geometry-aware reconstruction and texture refinement. The geometry-aware reconstruction proposes to exploit LF spatial-angular, location and geometry information to suppress occlusion issue. While the texture refinement stage aims to improve LF reconstruction quality by fully fusing LF spatial-angular, location and geometry information.

Experimental results illustrates the superiority of the proposed method than other state-of-the-art methods in improving BD-PSNR and reducing BD-Rate. Furthermore, the reconstruction quality comparisons and the application on depth estimation also validate that the proposed geometry-aware view reconstruction network can synthesize high-quality dense LF.

## Data Availability

Te datasets generated or analysed during the current study are available from the corresponding author on reasonable request.
